# Spontaneous Splenic Rupture Following Vaginal Delivery in Severe Preeclampsia: A Case Report

**DOI:** 10.7759/cureus.50266

**Published:** 2023-12-10

**Authors:** Chirag Sharma, Hina Patel

**Affiliations:** 1 Obstetrics and Gynaecology, Gujarat Medical Education and Research Society (GMERS) Medical College and Hospital, Valsad, IND

**Keywords:** high risk obstetrics, postpartum complication, explorative laparotomy, spontaneous splenic rupture, severe preeclampsia

## Abstract

Spontaneous rupture of the spleen during pregnancy is a rare and life-threatening occurrence, typically occurring in the third trimester or postpartum period. The mechanisms behind this phenomenon are still not fully understood, as it can happen without any obvious trauma, and even a minor abdominal strain can trigger it. We present a case of a 25-year-old woman with severe preeclampsia in which vaginal delivery was followed by spontaneous splenic rupture. A splenectomy was performed. Early diagnosis and management are crucial and can be aided by physical examination, ultrasonography, and clinical suspicion. It is imperative for obstetricians to be aware of this potentially fatal condition, as delayed diagnosis and treatment can have serious consequences for both the mother and the neonate.

## Introduction

Spontaneous rupture of the spleen during pregnancy is infrequent, usually occurring in the third trimester or postpartum period [1]. Initially considered rare, the growing number of reported cases in case reports and series indicates that this condition may be more prevalent than previously thought [2]. It is typically associated with underlying pathological conditions, including preeclampsia [1]. This complication often arises from pre-existing spleen conditions, such as splenic artery aneurysm or thalassemia, or from infectious causes such as malaria, typhoid, or infectious mononucleosis [3].

A spontaneous splenic rupture is defined as one that occurs without any associated trauma, in the absence of systemic diseases affecting the spleen, and when there is no evidence of perisplenic adhesions suggestive of prior trauma during laparotomy. Additionally, the spleen should appear normal both macroscopically and microscopically [3]. During pregnancy, splenic rupture is considered a serious condition associated with high maternal and fetal mortality and morbidity. Owing to its nonspecific symptoms, the diagnosis is often delayed, leading to unfavorable outcomes [1].

In this case report, we present a case of severe preeclampsia in which vaginal delivery was followed by spontaneous splenic rupture.

## Case presentation

A 25-year-old woman, gravida 4, para 3, living 3, with a gestational age of 38 weeks and 4 days based on her last menstrual period, was transferred to our facility from a secondary healthcare unit with a diagnosis of severe preeclampsia in the latent phase of labor. She reported experiencing epigastric pain for one day but denied having a fever, vomiting, blurred vision, or headaches. There were no significant findings in her past, medical, or family history. Her obstetric records indicated three prior successful full-term vaginal deliveries, occurring five, three, and two years ago, which resulted in the birth of one healthy male and two female infants.

Upon physical examination, the patient presented with symptoms of anxiety but was well-oriented. Her temperature was measured at 37.3°C, and she exhibited mild pallor and bilateral pitting pedal edema. Initial blood pressure was recorded at 170/100 mmHg, with a pulse rate of 84 and a respiratory rate of 14. Upon catheterization of the bladder, clear urine was drained. Abdominal examination revealed a uterus of 36 weeks' size with clearly audible fetal heart sounds. Cervical examination indicated a short, soft cervix that was two fingers loose. The patient delivered a healthy male infant weighing 2.8kg four hours after admission to the facility. Following delivery, the patient experienced uterine atony and subsequently developed primary postpartum hemorrhage, which was promptly managed through fundal massage and intravenous oxytocin administration. The infusion of oxytocin was maintained for an additional four hours, with positive results. The patient was administered oral labetalol, magnesium sulfate following Pritchard's protocol, and intravenous ceftriaxone at a dosage of 2 grams twice daily to provide antimicrobial coverage.

**Figure 1:**
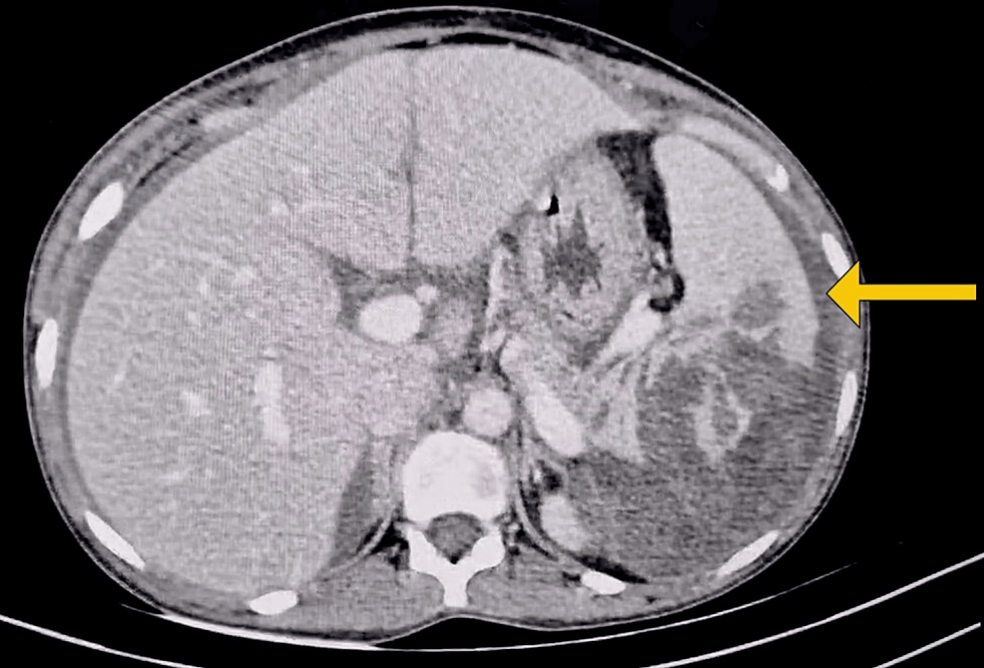
CECT of abdomen Splenic rupture with hemoperitoneum (indicated by a yellow arrow) CECT: Contrast-enhanced computed tomography

Five hours following childbirth, the mother exhibited tachycardia and hypotension, with a decrease in her hemoglobin levels from 10.2 g/dL pre-delivery to 7.9 g/dL. Subsequently, she underwent resuscitation and received 2 units of packed red blood cells (PRBC). Two hours following the transfusion of PRBC, the mother exhibited restlessness, tachycardia, and hypotension, accompanied by increasingly severe chest and abdominal pain. She displayed moderate pallor, with a pulse rate of 110 beats per minute of low volume and a blood pressure of 100/60 mmHg. Her respiratory rate was 22 breaths per minute. A physical examination revealed a distended, tense, and tender abdomen with guarding and rigidity. It was observed that her hemoglobin level had decreased from 7.9 g/dL to 5.6 g/dL. Emergent ultrasonography revealed a postpartum enlarged uterus, hepatomegaly, and a spleen with an irregular contour and some hypoechoic areas. Free fluid with moving internal echoes was observed in the peri-hepatic region, peri-splenic region, hepatorenal pouch, bilateral paracolic gutters, and pelvis. A contrast-enhanced computed tomography scan confirmed splenic rupture and significant hemoperitoneum (Figure 1).

**Figure 2:**
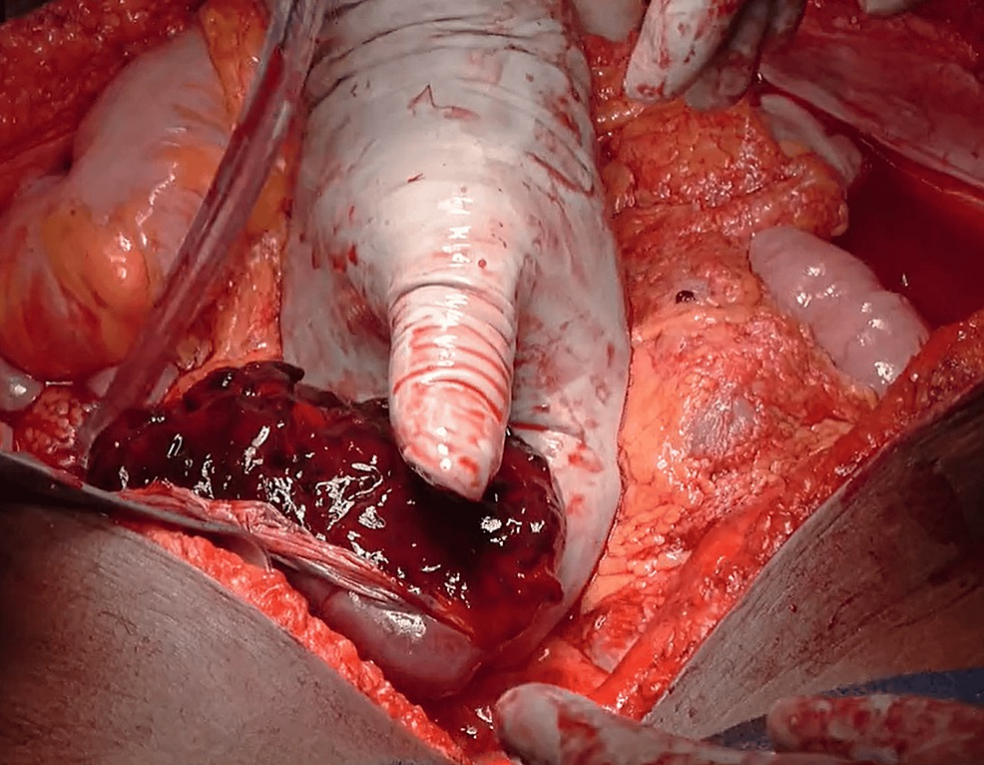
Spontaneous splenic rupture A defect in the splenic capsule near the hilum was identified on the superior surface

The patient was promptly transferred to the surgical intensive care unit. A committed interdisciplinary group of anesthetists, surgeons, and obstetricians was assembled. Following the resuscitation, the patient underwent an emergency laparotomy, during which the abdomen was opened with a midline vertical incision. Hemoperitoneum was confirmed during the procedure, and 2.5 liters of blood were drained. No uterine rupture was observed, and the bilateral ovaries and fallopian tubes appeared healthy. A significant accumulation of blood clots, weighing approximately 750 grams, was found in the upper left abdomen near the peri-splenic area. Upon removal of the clots, a 2.5x2 cm defect in the splenic capsule near the hilum was identified on the superior surface. Subsequently, a splenectomy was performed (Figure 2).

An intraperitoneal drain was maintained to monitor for continued bleeding. A total of six units of PRBC and six units of fresh frozen plasma were administered. Following the surgery, her vital signs were monitored in the obstetric intensive care unit. The postoperative recovery was without any complications. She was discharged on the 15th day after the surgery with a recommendation to take hematinic supplements.

## Discussion

Spontaneous splenic rupture during pregnancy in the absence of prior trauma is a rare occurrence, most commonly occurring in the third trimester or postpartum period. Among the 89 documented cases of splenic rupture during pregnancy, only 2.2% were reported to be spontaneous in the postpartum period [1]. Cases of splenic rupture during early pregnancy have been associated with splenic ectopic pregnancy, as well as in late pregnancy with preeclampsia, attributed to endothelial damage and thrombosis [4]. Spontaneous splenic rupture should be considered in cases where all other potential causes have been excluded, such as antecedent trauma, systemic disease, or evident abnormality upon examination. Similarly, the spleen's parenchyma, vasculature, and capsule should exhibit normal macroscopic and histological characteristics. Classic indicators of spontaneous splenic rupture encompass abdominal pain, left shoulder pain, and shock [1].

Potential mechanisms for this phenomenon may involve repeated spleen torsion due to heightened motility, collateral drainage obstruction or portal vein thrombosis, and splenic vein spasm leading to congestion [1]. The spleen becomes increasingly delicate as a consequence of vascular congestion caused by elevated plasma and erythrocyte levels resulting from a substantial rise in maternal blood volume, particularly in cases of twin pregnancies and during the third trimester [4]. Spontaneous rupture of the splenic capsule may occur due to increased volume, delivery trauma, and congenital factors such as a short splenic pedicle or deeply recessed location of the spleen. This can result from compression of the diaphragm during activities such as coughing, sneezing, or vomiting [1]. Furthermore, rapid plasma expansion with blood products and other volume expanders may also predispose the spleen to rupture due to a sudden increase in volume [2]. Postpartum spontaneous splenic rupture, particularly in individuals with high blood pressure, may be induced by abdominal packing and forceful tractions during cesarean sections [1]. During cesarean delivery or the removal of clots from the paracolic gutters, the application of excessive force while exploring the upper abdomen or manually expressing the fetus through forceful pushing may result in splenic injury [2].

The potential reasons for spontaneous splenic rupture in the general, nonpregnant population encompass various factors, including local splenic disorders, hematologic diseases, metabolic disorders, drug-induced and iatrogenic causes, and infectious causes like infectious mononucleosis. Infectious mononucleosis, in particular, is recognized as the most prevalent reason for spontaneous splenic rupture, along with conditions such as malaria [1]. An economical and convenient method for promptly diagnosing intraperitoneal fluid accumulation or hematoma is abdominal ultrasound, which can be conveniently conducted at the patient's bedside or in the emergency unit. This proves especially valuable in the initial assessment of patients experiencing hemodynamic instability and abdominal distention, particularly when considering exploration or when computed tomography is not a viable option [2].

The management of splenic rupture is dependent on various factors, such as the patient's clinical condition, the underlying pathology, and the extent of the injury [4]. In cases of spontaneous postpartum splenic rupture, emergency splenectomy is the established treatment protocol [2]. However, for patients who are being considered for nonoperative management, certain criteria must be met, including stable hemodynamics, the absence of peritoneal signs or other abdominal injuries that require surgery, the absence of preexisting splenic disease, an age below 55 years, a lower-grade injury, and minimal hemoperitoneum. Another option is splenic artery angiography followed by embolization, which has shown an 85% success rate [2].

The removal of the spleen raises the likelihood of regular infections and a higher overall risk of illness and death. Therefore, it is important for patients to adhere to vaccination measures for prevention. Compliance with vaccine prophylaxis is crucial [4]. The documented maternal mortality from splenic rupture varies from 0% to 45%, and there is a 47-82% chance of fetal loss [2].

## Conclusions

It is important to consider the differential diagnosis of spontaneous splenic rupture in pregnant women with severe preeclampsia due to the associated severe complications. If a woman experiences severe abdominal pain and distention with unstable vitals following a minor abdominal strain or trauma, along with or without vaginal bleeding, it may indicate splenic rupture in the postpartum period. Early diagnosis can be facilitated through physical examination and ultrasonography. It is crucial to recognize spontaneous postpartum splenic rupture as a surgical emergency that requires immediate attention, as delayed diagnosis can result in catastrophic consequences.
